# Supplementary material for: A feed-forward loop between SorLA and HER3 determines heregulin response and neratinib resistance
Source: Oncogene. 2021 Jan 8;40(7):1300–17. doi: 10.1038/s41388-020-01604-5 (PMC7892347; doi:10.1038/s41388-020-01604-5)
Supplement: Supplementary file 1 — Supplementary information [file 41388_2020_1604_MOESM1_ESM.pdf]

Figure S1

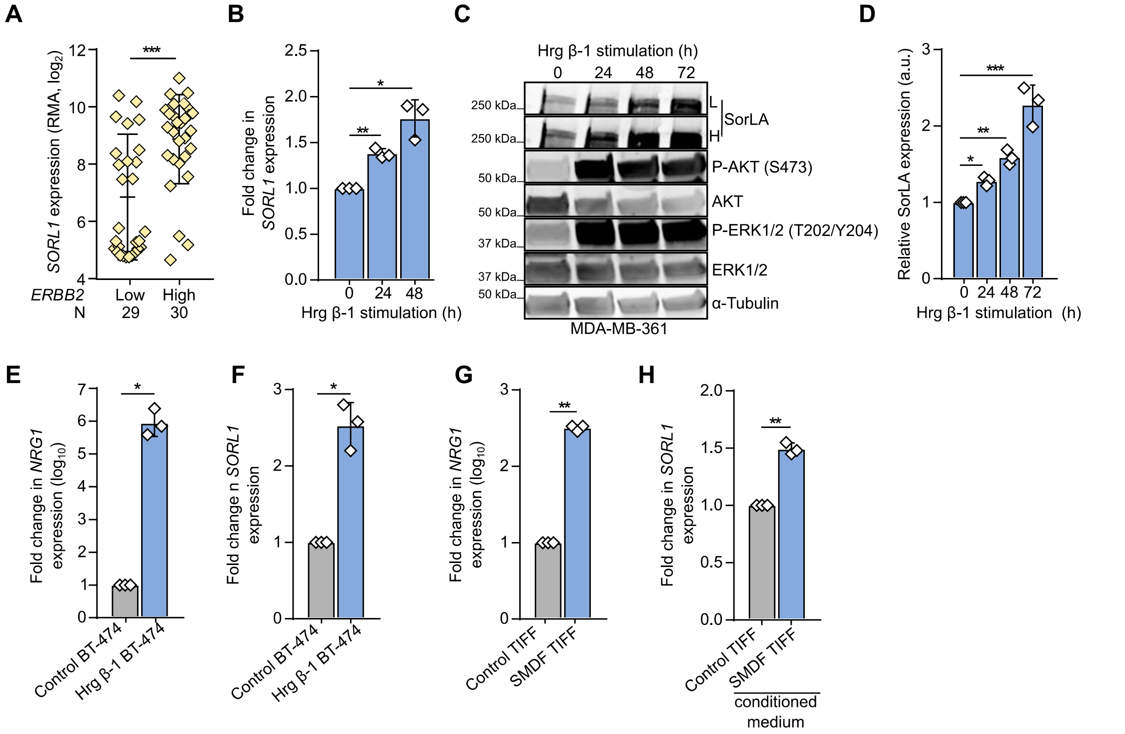

HER3 signaling regulates *SORL1* expression. **A**. *SORL1* expression is significantly higher in breast cancer cell lines with high *ERBB2* expression (CCLE; N=59). Data are mean ± SD; statistical analysis: Mann-Whitney U. **B**. MDA-MB-361 cells were stimulated with 20 ng.mL^{-1} Hrg β-1 for the indicated times. Quantification of *SORL1* mRNA levels, normalized to *HPRT1*, determined with RT-qPCR relative to non-stimulated (0 h) cells. **C**. MDA-MB-361 cells were stimulated with 20 ng.mL^{-1} Hrg β-1 for the indicated times. Representative immunoblotting of SorLA, AKT(p)S473, total AKT, ERK1/2(p)T202/Y204, total ERK1/2, with α-tubulin as a loading control. **D**. Quantification of SorLA levels normalized to loading control and relative to non-stimulated (0 h) cells. **E and F**. Quantification of *NRG1* (**E**) or *SORL1* (**F**) mRNA levels, normalized to *HPRT1*, determined with RT-qPCR in mCherry control or Hrg β-1-expressing BT-474 cells relative to control cells. **G.** Quantification of *NRG1* mRNA levels, normalized to *HPRT1*, determined with RT-qPCR in mCherry control or SMDF-overexpressing fibroblasts (TIFF) relative to control cells. **H**. Quantification of *SORL1* mRNA levels, normalized to *HPRT1*, determined with RT-qPCR in BT-474 cells after 24 h of culture with conditioned media from either control or SMDF-TIFF relative to control TIFF.

Unless otherwise indicated, Data are mean ± SD from three independent biological experiments; statistical analysis: Student's t-test (unpaired, two-tailed, unequal variance).

Figure S2

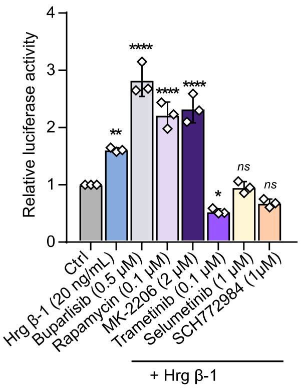

Heregulin-induced *SORL1* regulation requires HER3 signaling through ERK1/2. Inhibitors of the ERK pathway decrease Hrg β-1-induced luciferase activity. P3-Luc was expressed in BT-474 cells together with pRL-TK Renilla luciferase transfection control and cells were treated with 20 ng.mL^{-1} Hrg β-1 and the indicated inhibitors for 24 h. Luciferase activities are represented as fold increase from basal P3 luciferase activity. Data are mean ± SD from three independent biological experiments; statistical analysis: One-way ANOVA, Dunn's multiple comparisons test.

Figure S3

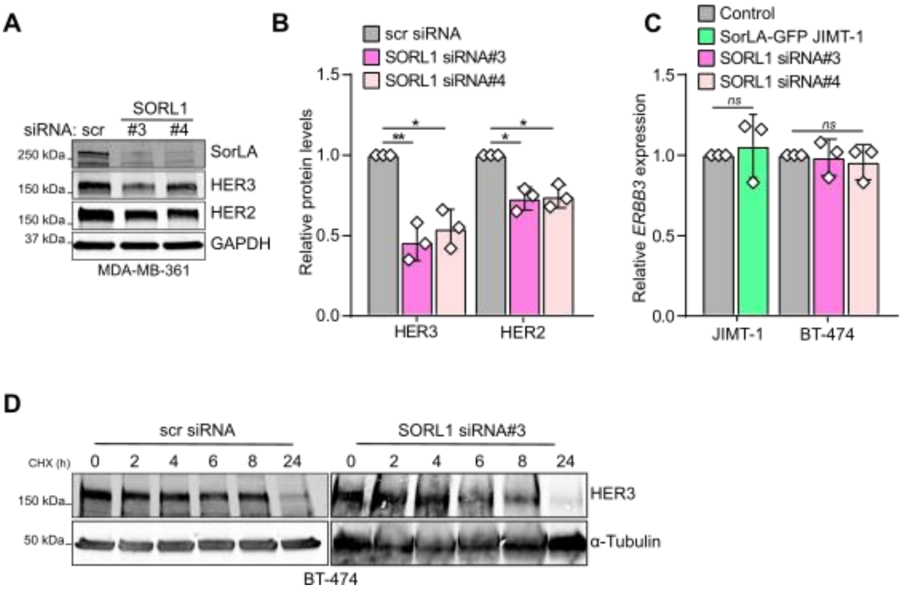

SorLA regulates HER3 stability. **A**. Representative immunoblotting of HER2, HER3 and SorLA in control (Scr) and SorLA RNAi transfected MDA-MB-361 cells, with

GAPDH as a loading control. **B**. Quantification of HER2 and HER3 levels normalized to loading control and relative to control-silenced cells. **C**. Quantification of *ERBB3* mRNA levels, normalized to *HPRT1*, determined with RT-qPCR in the cell lines transfected as indicated and relative to GFP-transfected controls (JIMT-1) or control siRNA-transfected BT-474 cells. **D**. Representative immunoblotting of HER3 in SorLA-silenced and control-silenced BT-474 cells treated with CHX for the indicated time points, with α-tubulin as a loading control. Double amount of protein was loaded for SorLA-silenced cells to allow equal detection of HER3.

Figure S4

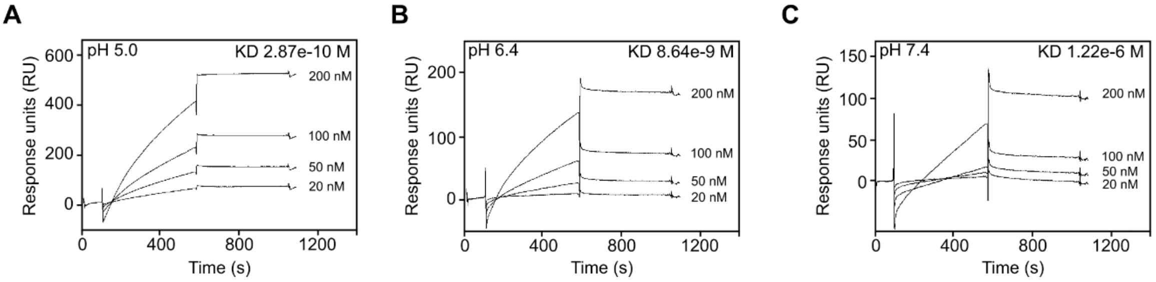

SorLA interacts with HER2-HER3 dimers. SorLA interacts with HER2 in a pH-dependent manner. SPR analysis of immobilized SorLA over a 20-200 nM concentration series of HER2 at pH 5.0 (**A**), 6.0 (**B**) and 7.4 (**C**).

Figure S5

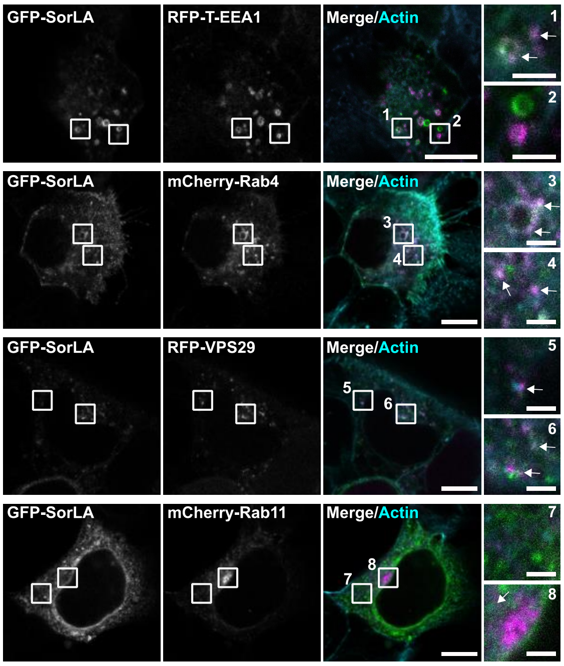

SorLA regulation of HER2 and HER3 requires functional Rab4. Representative confocal microscopy images of BT-474 cells co-expressing GFP-SorLA with the indicated endosomal markers. SiR-Actin was used for counterstaining the actin cytoskeleton. White arrows depict co-localizing signals. Scale bars: 10 µm. Scale bars (insets): 2 µm.

Supplementary Table 1: information related to the antibodies used in this study.

Antibody	Manufacturer	Catalogue No.	Dilution
HER3/ErbB3 (D22C5)	Cell Signaling Technology	#12708	1:1000
p-AKT (S473)	Cell Signaling Technology	#9271S	1:1000
Total AKT	Cell Signaling Technology	#9272	1:1000
phospho-ERK p-p44/42 MAPK T202/Y204	Cell Signaling Technology	#4370S	1 :1000
Total ERK p44/p42 MAPK	Cell Signaling Technology	9102S	1:1000
LR11 (SORL1)	BD Transduction Lab	612633	1:1000

HER2/ErbB2 (e2-4001 + 3B5)	Thermo Scientific	MA5-14057	1:1000
GAPDH	HyTest	5G4MaB6C5	1:2000
α-tubulin	Hybridoma Bank	12g10	1:5000
β-actin	Sigma	A1978	1:5000

Supplementary Table 2: list of previously published plasmids used in this study.

**Plasmid**	**Reference**
GFP-SorLA	(1)
RFP-T–EEA1	(2)
RFP-VPS29	(3)
mCherry-Rab4	A gift from Michael Davidson (Addgene plasmid # 55125)
mCherry-Rab11	A gift from Michael Davidson (Addgene plasmid # 55124)
GFP-Rab4^{S22N}	(4)

Supplementary Table 3: kinetics of SorLA interaction with HER2 and HER3 calculated using BIAEVALUATION 4.1 software.

Receptors	pH	ka (1/Ms)	kd (1/s)	KD (M)
SorLA:HER2	5.0	2.135e4	6.126e-6	2.869e-10
	6.4	4.703e4	4.061e-4	8.635e-9
	7.4	1.866e3	2.284e-3	1.224e-6
SorLA:HER3	5.0	7.088e4	2.871e-4	4.050e-9
	6.0	1.613e4	1.088e-4	6.745e-9
	7.4	2.160e5	2.075e-3	9.606e-9

References

1. Pietilä M, Sahgal P, Peuhu E, Jäntti NZ, Paatero I, Närvä E, et al. SORLA regulates endosomal trafficking and oncogenic fitness of HER2. Nat Commun. 2019 28;10(1):2340.

2. Navaroli DM, Bellvé KD, Standley C, Lifshitz LM, Cardia J, Lambright D, et al. Rabenosyn-5 defines the fate of the transferrin receptor following clathrin-mediated endocytosis. Proc Natl Acad Sci U S A. 2012 Feb 21;109(8):E471-480.

3. Hesketh GG, Pérez-Dorado I, Jackson LP, Wartosch L, Schäfer IB, Gray SR, et al. VARP Is Recruited on to Endosomes by Direct Interaction with Retromer, Where Together They Function in Export to the Cell Surface. Dev Cell. 2014 Jun 9;29(5):591–606.

4. Arjonen A, Alanko J, Veltel S, Ivaska J. Distinct Recycling of Active and Inactive β1 Integrins. Traffic Cph Den. 2012 Apr;13(4):610–25.
